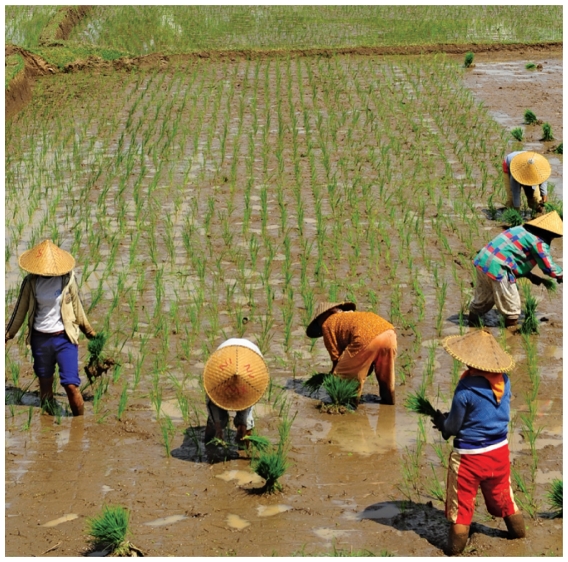# The Beat

**Published:** 2010-09

**Authors:** Erin E. Dooley

## Asian Tiger Mosquitoes Roar Indoors

A new study from Penang Island, Malaysia, finds that the Asian tiger mosquito (*Aedes albopictus*) is adapting to indoor environments, a factor that could increase vector–host contacts and the population density of the vector, thereby potentially increasing the diseases spread by this vector.^1^ The study showed the indoor-adapted mosquitoes had a longer lifespan and completed more reproductive cycles than outdoor-breeding mosquitoes. Asian tiger mosquitoes spread dengue viruses, chikungunya, yellow fever, and encephalitis viruses. These mosquitoes are linked to a rare U.S. outbreak of dengue fever in May 2009.

**Figure:**
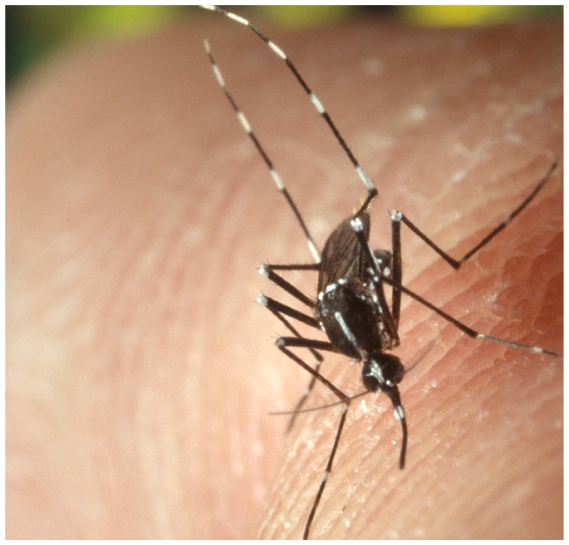


## New Cigarette Label Regulations Take Effect

Although consumers may assume cigarettes labeled *light*, *low*, or *mild* are healthier than regular cigarettes, there is no substantial scientific evidence that proves low-tar cigarettes cause fewer smoking-related health effects. Since 22 July 2010 those labeling terms have become off limits to cigarettes distributed in the United States under the Family Smoking Prevention and Tobacco Control Act of 2009.^2^ By July 2011 the U.S. FDA will establish requirements for large cigarette health warnings on labels, including color graphics depicting the adverse health effects of smoking, says FDA representative Kathleen Quinn.

**Figure:**
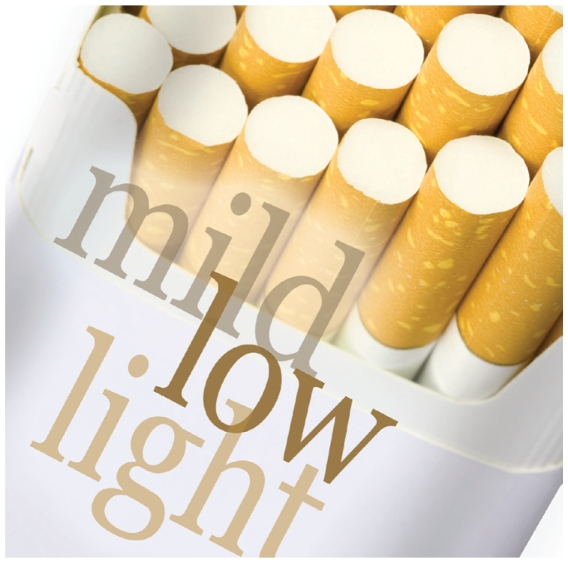


## Light-Colored Roofs Cool Cities

Roofs and pavements cover 50–65% of urban areas. Using a detailed NASA global land surface model, researchers have found that light-colored rooftops and road surfaces can offset the heating effect of up to two years of current global CO_2_ emissions.^3^ The increased reflectivity of these surfaces allows entire buildings and surrounding areas to maintain cooler temperatures. The result: less energy is required to keep indoor temperatures comfortable. The study looked at the effect of changing urban surfaces to cooler colors, not just bright white—a color that may not appeal to some homeowners. In July 2010, DOE Secretary Steven Chu announced a federal initiative under President Obama’s Executive Order on Sustainability to implement cool roof technologies on government facilities.^4^

## Stronger Ozone Layer Protection May Reduce Cataract Incidence

A new EPA report indicates stricter 1997 amendments to the Montreal Protocol may be paying off.^5^ The report predicts more than 22 million additional cataract cases may be avoided in Americans born between 1985 and 2100 thanks to successful ongoing efforts to repair the Earth’s ozone layer. The EPA report used the recently updated Atmospheric and Health Effects Framework model to predict avoided cataract cases. According to the U.S. EPA, the ozone layer is expected to recover to pre-1980 levels by 2065.

## Hotter Nights May Affect Asian Rice Crops

A six-year study, the first of its kind using real-world data, shows that hotter nights affect rice productivity, and that with continued climate change the effect may worsen as this century progresses.^6^ The authors found increased nighttime temperatures affected a key stage in ripening known as grain filling—perhaps because energy the rice usually spent ripening was diverted to increased respiration. They predict the scenario may get worse as daytime temperatures increase to certain predicted levels, which also may restrict the growth cycle of rice plants.

**Figure:**